# Supplementary material for: Inhibition of Granuloma Triglyceride Synthesis Imparts Control of Mycobacterium tuberculosis Through Curtailed Inflammatory Responses
Source: Front Immunol. 2021 Sep 15;12:722735. doi: 10.3389/fimmu.2021.722735 (PMC8479166; doi:10.3389/fimmu.2021.722735)
Supplement: Supplementary file 1 [file DataSheet_1.pdf]

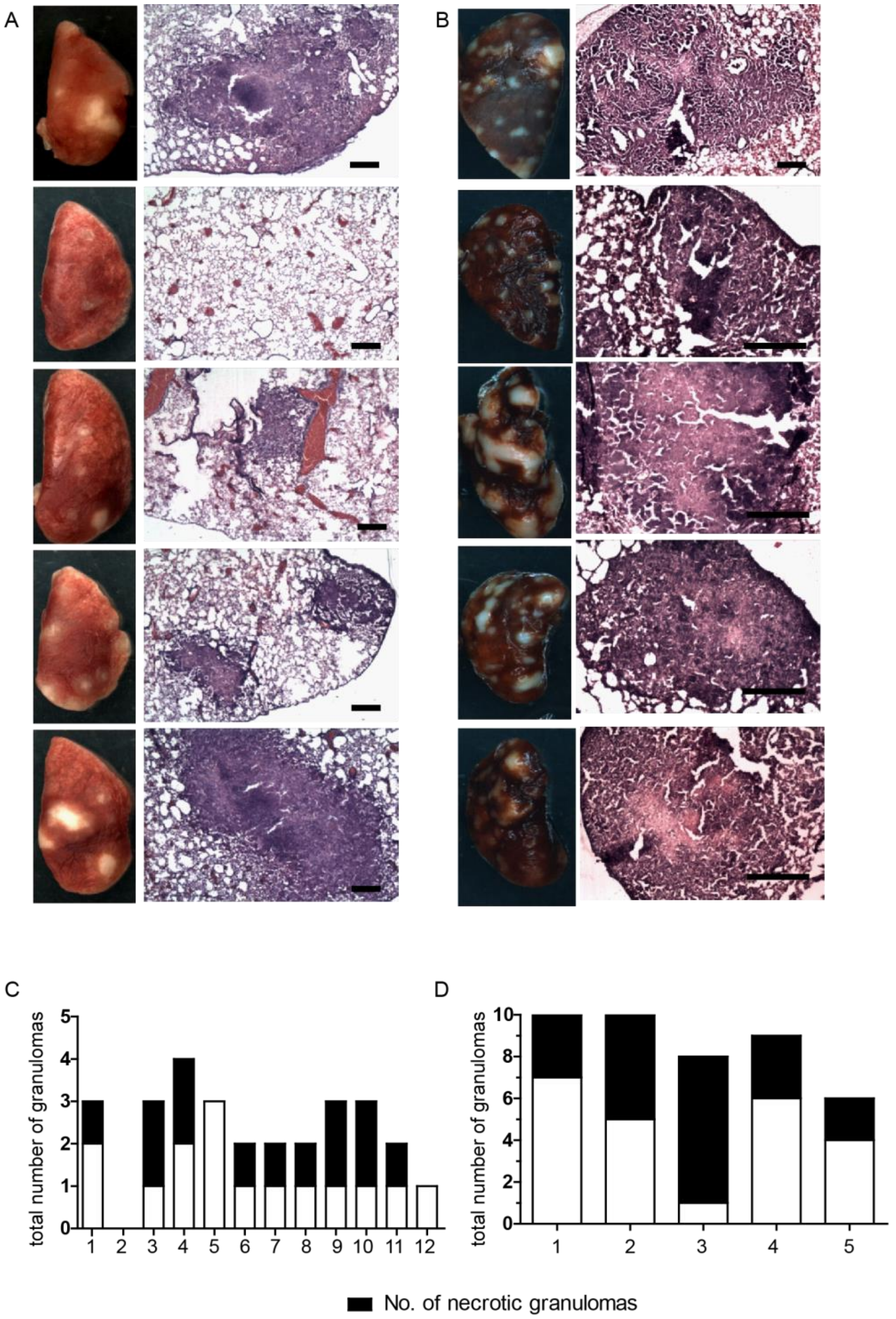

Figure S1. Gross pathology and cryosections from C3HeB/FeJ mice at d28 post infection with low dose (100 cfu) (A) and high dose (500 cfu) (B) of Erdman strain delivered via aerosol route. Section are stained with hematoxylin and eosin; scale bar=400 μm. Data are of 5 representative from low cfu and all 5 fromhigh CFU. Quantification of necrotic and total number of granulomas scored in the left caudal lobe of animals of low dose infection (C) and high dose of infection (D).

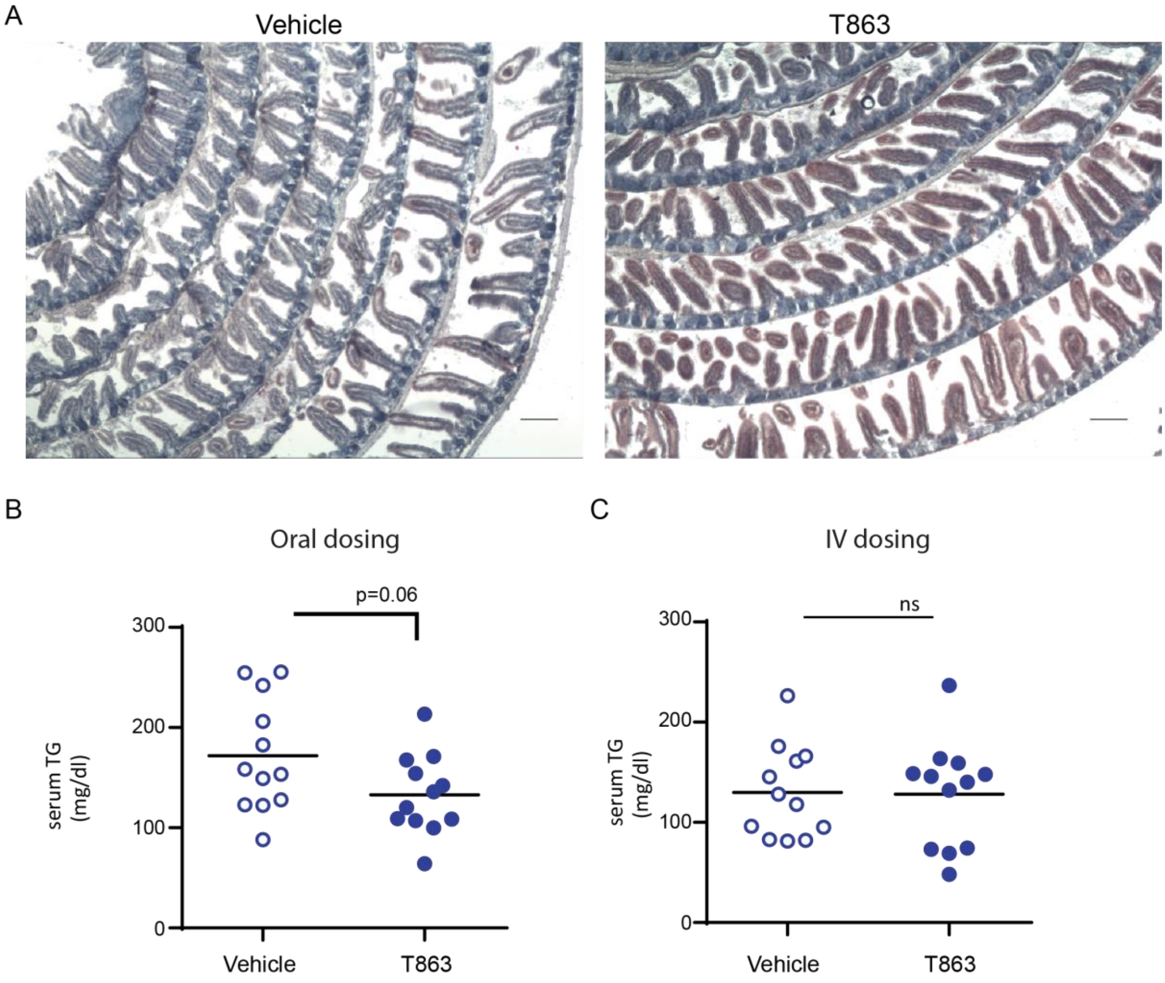

Figure S2. Effects of oral versus iv dosing of T863 on gut and serum TG levels. (A) Cyrosections of distal small intestine stained with oil red O and hematoxylin scale bar=200 μm. (B-C) Serum triglycerides in vehicle or T863 treated upon 3 weeks of oral (B) or intravenous (C) dosing.

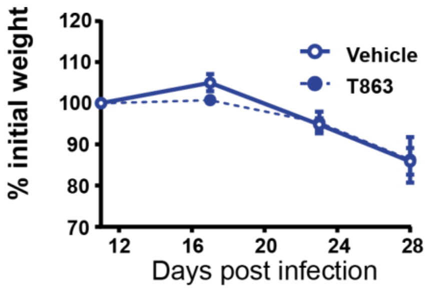

Figure S3. Effect of T863 treatment on weight loss over 3 weeks of treatment

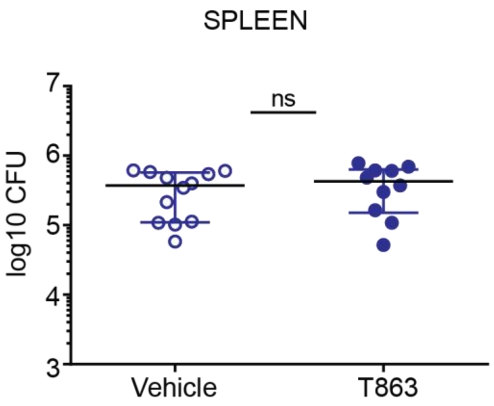

Figure S4. Splenic CFU at d28 from animals treated with Veicle or T863 for 3 weeks.

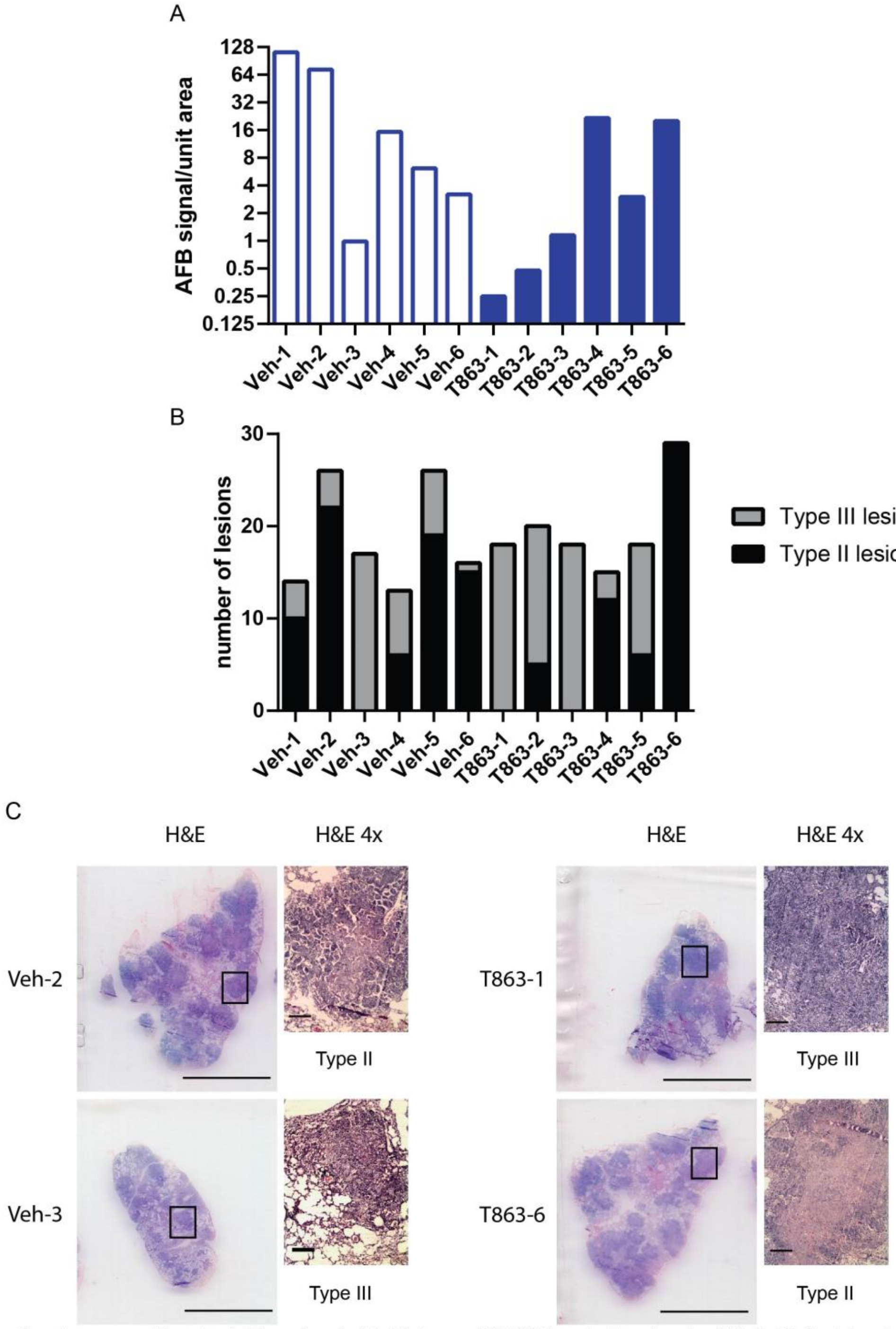

Figure S5. Lesion types and bacterial burden in Vehicle and T863 treated animals. (A) Acid-fast bacteria signal/ unit area in all the vehicle and T863 treated animals. (B) Total number of lesions and the number of type II and type III lesions present in all the animals. (C) Representative images from vehicle and T863 group which had developed type II and type III lesions, (C) The left panel shows the H&E stained whole lung sections and right panel shows the 4x magnification images of individual granulomas from the indicated region from the left panel (black box). Scale bar in the whole lung section= 5mm and scale bar in the 4x magnification image= 200 μm
